# Characterization of Cardiac-Resident Progenitor Cells Expressing High Aldehyde Dehydrogenase Activity

**DOI:** 10.1155/2013/503047

**Published:** 2013-01-31

**Authors:** Marc-Estienne Roehrich, Albert Spicher, Giuseppina Milano, Giuseppe Vassalli

**Affiliations:** ^1^Department of Cardiology, Centre Hospitalier Universitaire Vaudois (CHUV), Avenue du Bugnon, 1011 Lausanne, Switzerland; ^2^Department of Cardiovascular Surgery, Centre Hospitalier Universitaire Vaudois (CHUV), Avenue du Bugnon, 1011 Lausanne, Switzerland; ^3^Molecular Cardiology Laboratory, Fondazione Cardiocentro Ticino, Via Tesserete 48, 6900 Lugano, Switzerland

## Abstract

High aldehyde dehydrogenase (ALDH) activity has been associated with stem and progenitor cells in various tissues. Human cord blood and bone marrow ALDH-bright (ALDH^br^) cells have displayed angiogenic activity in preclinical studies and have been shown to be safe in clinical trials in patients with ischemic cardiovascular disease. The presence of ALDH^br^ cells in the heart has not been evaluated so far. We have characterized ALDH^br^ cells isolated from mouse hearts. One percent of nonmyocytic cells from neonatal and adult hearts were ALDH^br^. ALDH^very-br^ cells were more frequent in neonatal hearts than adult. ALDH^br^ cells were more frequent in atria than ventricles. Expression of ALDH1A1 isozyme transcripts was highest in ALDH^very-br^ cells, intermediate in ALDH^br^ cells, and lowest in ALDH^dim^ cells. ALDH1A2 expression was highest in ALDH^very-br^ cells, intermediate in ALDH^dim^ cells, and lowest in ALDH^br^ cells. ALDH1A3 and ALDH2 expression was detectable in ALDH^very-br^ and ALDH^br^ cells, unlike ALDH^dim^ cells, albeit at lower levels compared with ALDH1A1 and ALDH1A2. Freshly isolated ALDH^br^ cells were enriched for cells expressing stem cell antigen-1, CD34, CD90, CD44, and CD106. ALDH^br^ cells, unlike ALDH^dim^ cells, could be grown in culture for more than 40 passages. They expressed sarcomeric **α**-actinin and could be differentiated along multiple mesenchymal lineages. However, the proportion of ALDH^br^ cells declined with cell passage. In conclusion, the cardiac-derived ALDH^br^ population is enriched for progenitor cells that exhibit mesenchymal progenitor-like characteristics and can be expanded in culture. The regenerative potential of cardiac-derived ALDH^br^ cells remains to be evaluated.

## 1. Introduction

Growing evidence suggests the adult heart may harbor resident stem and progenitor cells that participate in adaptive responses to myocardial injury, and possibly in cellular homeostasis under normal conditions. In most studies, the prospective isolation of cardiac stem cells has relied upon the use of specific antibodies that recognize cell-surface antigens expressed by stem cells in other tissues, particularly by hematopoietic stem cells (HSCs). Cardiac stem cells expressing stem cell antigen-1 (Sca-1) or the stem cell factor receptor, c-kit (CD117), have been described [1, 2]. A distinct population of cardioblasts expressing the transcription factor islet-1 (Isl-1) and entering fully differentiated cardiomyocyte lineages has also been identified [3, 4]. However, the expression of cell-surface epitopes can vary with the metabolic state of the cell and the experimental conditions used. Therefore, no individual cell-surface antigen marker definitely identifies a single entity of cardiac stem cell. Functional properties of tissue-resident stem cells that could be used for their prospective isolation, regardless of cell-surface marker expression, have been intensely searched for. It has been proposed that, irrespective of their lineal origin, stem cells may share common mechanisms to regulate self-renewal and differentiation, and that candidate “stemness” genes may serve as universal stem cell markers.

Cai et al. [5] proposed that high aldehyde dehydrogenase (ALDH) activity is one of a small set of common characteristics shared by stem cells among tissues (recently reviewed by Balber [6]). Few ALDH-bright (ALDH^br^) pluripotential cells, measured by Aldefluor stain, gave rise to all somatic and reproductive cell lineages in tunicates [7, 8]. ALDH is a cytosolic enzyme responsible for the oxidation of intracellular aldehydes. It plays important roles in oxidation of alcohol and vitamin A, and in chemoresistance to cyclophosphamide. ALDH superfamily is highly conserved across species [9]. Nineteen ALDH isoforms are known in human. ALDH^br^, side scatter-low (SSC^lo^) populations from murine and human bone marrow (BM) [10–13], umbilical cord blood (UCB) [14–19], cytokine-mobilized peripheral blood [20], and circulating blood cells [21, 22] are enriched in stem and progenitor cells. The ALDH^br^SSC^lo^ population includes virtually all human CD34^+^ and CD133^+^ cells that establish long-term, multilineage hematopoietic colonies in culture or long-term, multilineage xenografts in immunodeficient mice [16]. More recently, ALDH^br^ stem cells have been identified in nonhematopoietic systems, such as circulating endothelial progenitor cells (EPCs) [21, 22], neural stem cells [23, 24], human muscle precursor cells with high myogenic activity [25, 26], colonic stem cells [27], and mammary stem cells [28]. Moreover, ALDH^br^ cancer stem cells have been described in multiple types of cancer and shown to predict poor clinical outcomes in various contexts [27–29].

We therefore asked whether the postnatal heart harbors ALDH^br^ cells, as measured by Aldefluor stain. We found that the neonatal as well as the adult mouse heart harbors a relatively small population of ALDH^br^ cells (*≈*1% of all nonmyocytic cells present in the heart), which is enriched for cells expressing Sca-1 and other progenitor cell markers. ALDH^br^ cells, but not ALDH^dim⁡^ cells, adhered to plastic and grew in culture. The expanded population expressed sarcomeric *α*-actinin, a cardiac marker, along with mesenchymal stem cell (MSC) markers. It could be induced to differentiate along multiple mesenchymal cell lineages. These results suggest that ALDH may mark a population of cardiac-resident, MSC-like progenitor cells that possess superior *ex vivo* growth characteristics.

## 2. Methods

### 2.1. Mice and Cell Isolation

Neonatal (postnatal day 1), young adult (8 week-old), and aging (24 month-old) C57Bl/6 mice were purchased from Charles River Laboratories (France). Immediately after the sacrifice of the mice, the chest was opened, a canula was introduced into the left ventricular cavity, an incision was made in the right atrial wall, and the heart was perfused with heparinized PBS. The explanted heart was washed in PBS and cut into small pieces that were then placed in a Falcon tube (50 mL) containing 5 mL RPMI 1640 medium (Invitrogen, Carlsbad, CA, USA) supplemented with 12.5 *μ*L Liberase Blendzyme 4 and 25 *μ*L DNAse I (both from Roche, Basle, Switzerland), and incubated for 45 min at 37°C under gentle shaking. The cell suspension was filtered through a 70 *μ*m-filter using a syringe plunger, washed in PBS, and centrifuged. The pellet was resuspended in 100 *μ*L Dead Cell Removal Kit microbeads solution (Miltenyi, Bergisch Gladbach, Germany), incubated for 15 min at RT, changed to Dead Cell Removal Buffer, and passaged through a Miltenyi LS column. The flow-through was centrifuged and the pellet was resuspended in FACS buffer. In a subset of experiments, atria and ventricles were processed separately to measure ALDH^br^ cells in the different cardiac chambers of origin.

### 2.2. Flow Cytometric Analyses

Cells were washed in PBS, resuspended in Aldefluor buffer, and then reacted with Aldefluor substrate (STEMCELL Technologies, Vancouver, BC, Canada) according to the manufacturer's instructions. After the ALDH enzyme reaction, cells were washed, resuspended in cold Aldefluor buffer, and maintained on ice during all subsequent manipulations. A forward scatter (FSC) versus side scatter (SSC) cytogram was used to gate signals from cells, and an Aldefluor fluorescence versus SSC cytogram was constructed. A unique ALDH^br^ population was present in Aldefluor-reacted samples and absent in cells in the samples treated with the specific inhibitor of ALDH, diethylaminobenzaldehyde (DEAB). Cells incubated with Aldefluor substrate and DEAB were used to establish baseline fluorescence of these cells and to define the ALDH^br^ and the ALDH^very-br^ region as less than 0.1% and 0% of total events, respectively. Cell incubation with Aldefluor substrate in the absence of inhibitor induced a shift in FL1 fluorescence defining the ALDH^dim⁡^, the ALDH^br^, and the ALDH^very-br^ population. To assess cell-surface antigen expression, cells were incubated with antibody for 20 min, washed, and resuspended in cold Aldefluor buffer. Flow cytometric analyses were performed on a FACSCalibur instrument (Becton Dickinson, San Jose, CA, USA) operating at 488 nm excitation with standard emission filters. Aldefluor fluorescence was measured in FL1 and APC in FL4. Gates used to resolve antigen-expressing cells were set using appropriate isotype-matched control Abs. Data files containing at least 2,000 ALDH^br^ cells were acquired for analysis using the CellQuest software (Becton Dickinson).

### 2.3. Immunostaining

Culture-expanded ALDH^br^ cells grown in Lab-Tek chambers were fixed with 1% paraformaldehyde (PFA) for 10 min, followed by blocking solution (1x PBS, 1% BSA, 0,3% Triton) for 1 h, and incubated with rat anti-mouse c-Kit/CD117 mAb (clone 3C1; Miltenyi) coupled to APC for 1 h at RT, followed by goat anti-rat IgG coupled to Alexa 488 (1 : 400 dilution; Molecular Probes, Life Technologies, Grand Island, NY, USA). To detect type II collagen, cells were fixed in PFA, incubated in blocking solution for 1 h, and then with mouse monoclonal anti-type II collagen 4 Abs coktail (1 : 100 dilution; Chondrex, Redmond, WA, USA), followed by goat anti-mouse IgG coupled to Alexa 488 (1 : 400). To detect sarcomeric *α*-actinin, cells were fixed with 4% PFA for 1 h, incubated in blocking solution for 1 h, and then with mouse antisarcomeric *α*-actinin mAb (1 : 750 dilution; clone EA-53, Sigma-Aldrich, St. Louis, MO, USA) at 4°C overnight, followed by goat anti-mouse IgG coupled to Alexa 488 (1 : 400). To detect *α*-smooth muscle actin (*α*-SMA), cells were stained with anti-*α*-SMA rabbit polyclonal Ab (abcam5694; Abcam). Control sections were incubated with secondary Ab only. Nuclei were stained with Hoechst 33342 and slides were mounted with Mowiol. Pictures were taken with a Zeiss Axioplant 2 microscope (100x objective).

### 2.4. Antibodies

The following mAbs were used to detect the cell-surface antigen distribution of cardiac cells by flow cytometry: CD11b-APC (clone M1; Miltenyi), CD14-APC (clone Sa2-8; eBioscience; San Diego, CA, USA), CD29-PE (clone RTK2758; BioLegend), CD31-APC (clone 390; eBioscience), CD34-APC (clone RAM-34; eBioscience), CD38-APC (clone 90; eBioscience), CD40-APC (clone 1C10; eBioscience), CD44-APC (clone IM7; eBioscience), CD45-APC (clone 30-F11; eBioscience), CD90.2-APC (clone HIS51; eBioscience), CD105-PE (clone MJ7/18; eBioscience), CD106-PE (clone 429; eBioscience), c-Kit/CD117-APC (clone 3C1; Miltenyi), CD133-APC (clone 13A4; eBioscience), CD140b-APC (eBioscience), CD146-FITC (Miltenyi), Flk-1-APC (clone Avas12a1; eBioscience), Lineage Cell Detection Cocktail-Biotin mouse (Miltenyi; cat. no. 130-092-613), MHC class II-APC (clone M5; eBioscience), NG2 chondroitin sulfate proteoglycan (Chemicon/Millipore; cat. no. AB5320), Sca-1-APC (clone D7; eBioscience), rat IgG2a isotype control-APC (clone eBR2a; eBioscience), rat IgG2a isotype control-FITC (clone eBR2a; eBioscience), rat IgG2a isotype control-PE (clone eBR2a; eBioscience), rat mouse IgG1, *κ* isotype control (clone P3; eBioscience), and Arminian hamster IgG isotype control (clone eBio299Arm; eBioscience).

### 2.5. Real Time RT-PCR

Freshly isolated ALDH^dim⁡^, ALDH^br^, and ALDH^very-br^ cells from PBS-perfused hearts from 8-week-old mice (*n* = 4) were sorted using a Beckman Coulter MoFlo Astrios FACS system. Total mRNA from each cell subset was extracted using the RNeasy Micro kit (Qiagen). The different c-DNAs were generated using the Quantitect reverse transcription kit (Qiagen) from total mRNA obtained with a genomic DNA digestion step according to the manufacturer's instructions. cDNA (1 : 10 dilution) was used for quantification using the RT^2^ SYBR Green qPCR Kit (Qiagen) and the Rotor-Gene 2000 system (Qiagen) according to the manufacturer's instructions. Real-time PCR reactions (in triplicates) were set up in 10 *μ*L reaction volume with 5 *μ*L of RT^2^ SYBR Green mix, 0.4 *μ*L of 10 *μ*M RT^2^ qPCR Primer Assay, 3 *μ*L cDNA, and 1.6 *μ*L water. The polymerase was heat-activated for 10 min at 95°C, and the reactions were then cycled 50 times (95°C, 15 sec; 55°C, 40 sec; 72°C, 30 sec), followed by a melting step. Primers were obtained from sAbiosciences (Qiagen). Relative expression was calculated with the comparative ΔCt method using *GUSB* as a reference gene.

### 2.6. *Ex Vivo* Cultures of ALDH^br^ Cells

In two preliminary experiments, cells from enzymatically dissociated atria and ventricles were reacted separately with Aldefluor, and ALDH^br^ cells were sorted by FACS and placed in Corning Costar 6-well plates (Sigma) with no extracellular matrix protein coating. Ventricular ALDH^br^ cells grew poorly in culture, possibly as a result of a very long sorting procedure due to the scarcity of ALDH^br^ cells in the ventricular population. Therefore, atrial cells from 8-week-old mice were used in subsequent experiments (*n* = 3 per experiment), allowing for a marked abbreviation of the sorting procedure. ALDH^br^ cells were cultured in MesenCult medium (MesenCult MSC Basal Medium supplemented with serum-containing MesenCult MSC Stimulatory Supplements-Mouse; STEMCELL Technologies). ALDH^dim⁡^ cells were studied for comparison. To assess the impact of the culture medium on cell phenotype, ALDH^br^ sorted cells were also cultured in RPMI/FCS medium (RPMI 1640; Gibco, supplemented with 10% fetal calf serum). The AlamarBlue assay (Promega, Madison, WI, USA) was used to assess cell viability and growth. In a separate experiment, cells were cultured in the presence of imatinib (methanesulfonate salt, 0.1–10 *μ*M; LC Laboratories, Woburn, MA, USA), a inhibitor of receptor tyrosine kinases encoded by c-kit and platelet-derived growth factor receptor B (PDGFRB). Cells were incubated with imatinib for 42 h (in triplicates) before AlamarBlue was added. Absorbance was measured at 570 nm and 595 nm every hour during 6 hours, and AlamarBlue reduction was calculated.

### 2.7. Differentiation Potential of Expanded ALDH^br^ Cells

Cells sorted on the basis of high ALDH activity were cultured for 8 months in MesenCult, which was then replaced by NH ChondroDiff Medium (for 21 days), NH OsteoDiff Medium (7 days), or AdipoDiff Medium (14 days; all from Miltenyi). Chondrogenic differentiation was assessed by immunostaining for type II collagen. To assess osteogenic and adipogenic differentiation, cells were incubated with 1% PFA for 10 min, followed by 2% Alizarin Red solution for 5 min and Oil Red-O solution for 15 min, respectively, and by 3 PBS-washes.

## 3. Results

### 3.1. ALDH^br^ Cells in Neonatal and Young Adult Hearts

**Figure 1:**
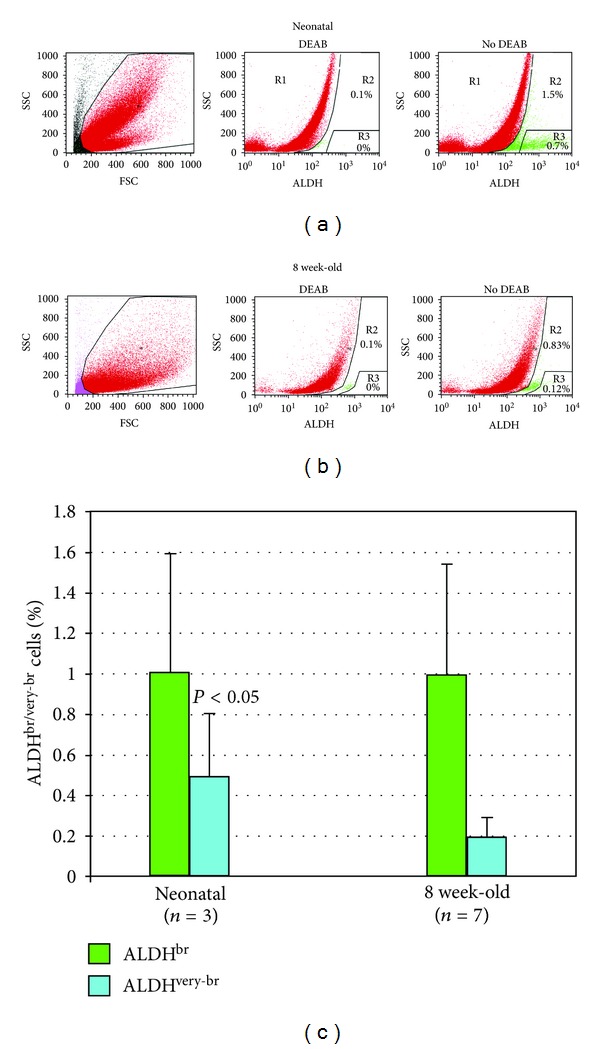
Identification of ALDH^br^ cells in populations isolated from whole hearts from neonatal and young adult mice. Flow cytometric analysis of ALDH activity (a). Neonatal mice. Left panel: cells were selected according to forward scatter (FSC) and side scatter (SSC) properties using the gated region. Middle panel: cells incubated with Aldefluor substrate and the specific inhibitor of ALDH, DEAB were used to establish baseline fluorescence of these cells and to define the ALDH^br^ (R2) and the ALDH^very-br^ (R3) region as less than 0.1% and 0% of total events, respectively. Right panel: cell incubation with Aldefluor substrate in the absence of inhibitor induced a shift in FL1 fluorescence defining the ALDH^dim⁡^ (R1), the ALDH^br^ (R2), and the ALDH^very-br^ (R3) populations (b). Eight-week-old mice (c). Bar histogram showing ALDH^br^ and ALDH^very-br^ cell percentages (mean, SD) in neonatal and young adult mice.  *P* value refers to the difference between neonatal and 8 week-old mice.

Freshly isolated nonmyocytic cells from enzymatically dissociated, neonatal or 8-week-old hearts were analyzed by flow cytometry using the Aldefluor reagent. ALDH^br^ cells amounted to 1.00 ± 0.59% of all nonmyocytic cells in the neonatal heart (*n* = 3) and to 0.99 ± 0.55% of these cells in the young adult heart (*n* = 7, NS; Figure 1). Within the ALDH^br^ population, the percentage of ALDH^very-br^ cells in neonatal hearts was higher than in young adults (52.71 ± 6.87% versus 19.39 ± 2.42%; *P* < 0.05).

### 3.2. ALDH^br^ Cells in Atria and Ventricles

**Figure 2:**
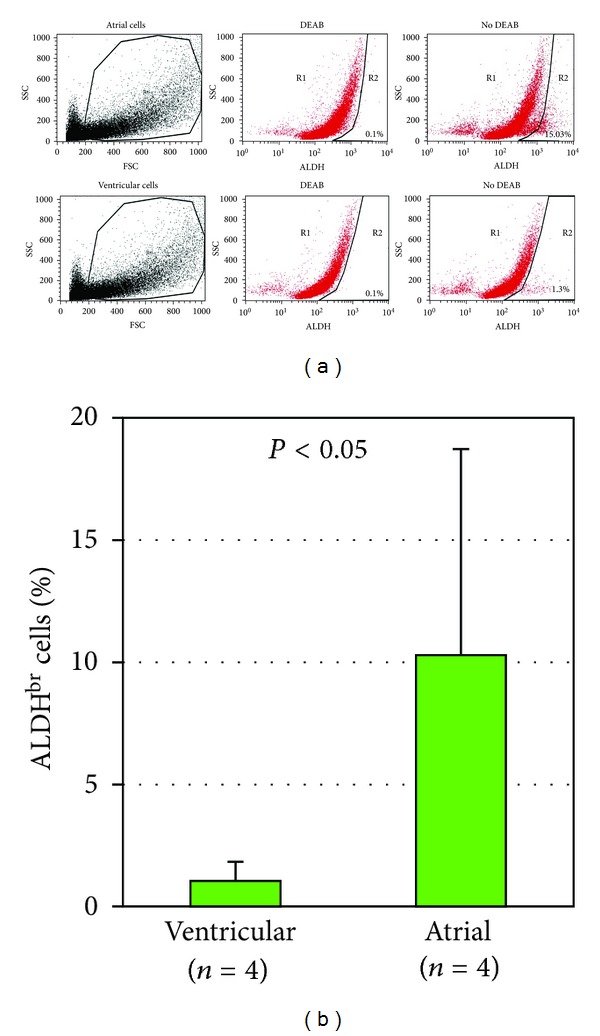
Identification of ALDH^br^ cells in the atrial and ventricular population from 8 week-old mice. Flow cytometric analysis of ALDH activity (a). Left panels: atrial and ventricular cells were selected according to forward scatter (FSC) and side scatter (SSC) properties using the gated region. Middle panels: cells incubated with Aldefluor substrate and the specific inhibitor of ALDH, DEAB, were used to establish baseline fluorescence of these cells and to define the ALDH^br^ region (R2) as less than 0.1% of total events. Right panels: cell incubation with Aldefluor in the absence of inhibitor induced a shift in FL1 fluorescence defining the ALDH^dim⁡^ (R1) and the ALDH^br^ (R2) population. In the example shown, not all of the atrial cells exhibiting a shift in FL1 fluorescence in the absence of inhibitor were found in the gated region R2, suggesting that atrial ALDH^br^ cells may have been underestimated (b). Bar histogram showing percent ALDH^br^ cells (mean, SD) in the ventricular and atrial population.

In young adult mice, ALDH^br^ cells in the atrial population were more frequent than in the ventricular (10.29 ± 8.44% versus 1.05 ± 0.78%; *n* = 4; *P* < 0.05; Figure 2). Atrial ALDH^br^ cells predominantly exhibited SSC^lo^ properties but a subset showed intermediate-to-high SSC properties. Ventricular ALDH^br^ cells exhibited more homogeneous SSC^lo^ properties. 

### 3.3. Immunophenotype of Freshly Isolated ALDH^br^ Cells

**Figure 3:**
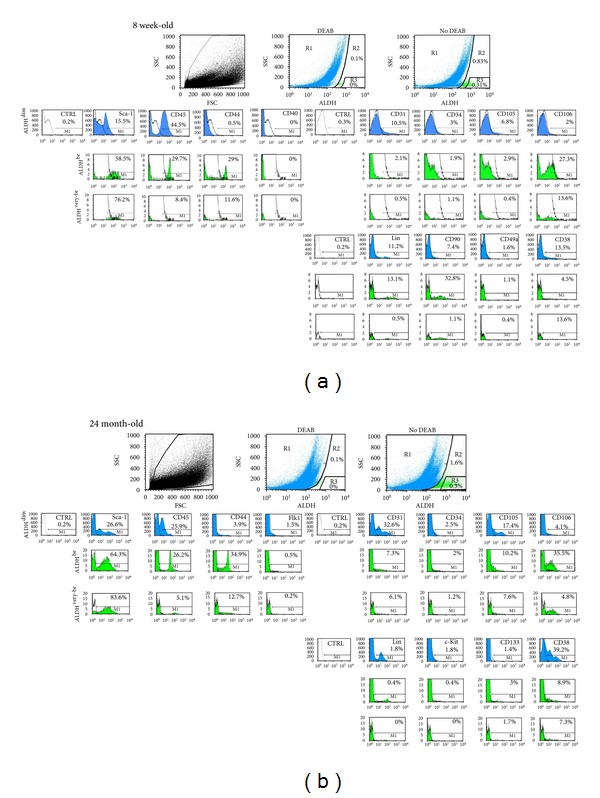
Freshly isolated ALDH^br^ cells from young adult hearts (upper panel; (a)) and from aging hearts (lower panel; (b)) were analyzed by flow cytometry for ALDH expression in combination with the expression of cell-surface markers. Cells were incubated with Aldefluor substrate and an inhibitor of ALDH (DEAB), or with Aldefluor alone, to establish R1, R2, and R3 gates for ALDH^dim⁡^ (blue), ALDH^br^, and ALDH^very-br^ cells (both green), respectively. These populations were subsequently analyzed for expression of markers. Cytograms for each surface marker in each cell population are shown (the surface markers indicated for the cytograms of ALDH^dim⁡^ cells also refer to the cytograms of the respective ALDH^br^ and ALDH^very-br^ populations; corresponding cytograms are aligned vertically. Percentages of positive cells are indicated (CTRL; isotype-matched irrelevant IgG).

**Figure 4:**
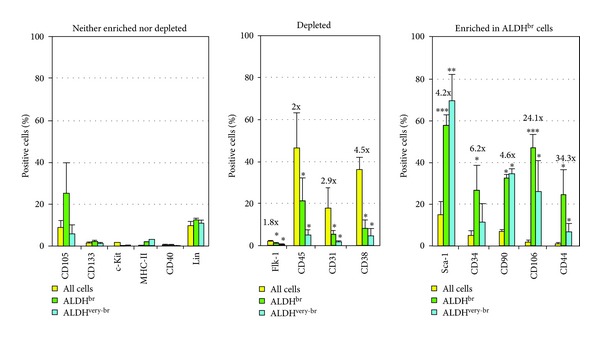
Flow cytometric analysis of the expression of cell-surface antigen markers in freshly isolated nonmyocytic cardiac cells from 8-week-old mice. Data are percentages (mean, SD) of positive cells for the indicated markers, subdivided into three groups: those significantly enriched, those significantly depleted, and those neither enriched nor depleted in the ALDH^br^ population. Yellow bars indicate bulk populations of cardiac-derived cells, green bars indicate ALDH^br^ cells, and blue bars indicate ALDH^very-br^ cells. Numbers are enrichment/depletion factors for the indicated surface markers in the ALDH^br^ population (***P < 0.001, *P < 0.05 versus ALDH^dim⁡^ cells).

We used a panel of mAbs to determine the cell-surface marker profile of ALDH^br^, ALDH^very-br^, and ALDH^dim⁡^ cells in the freshly isolated, nonmyocytic cardiac-derived population in 8 weeks old as well as in 24 months old mice (Figure 3). Aging hearts contained 1.36%   ALDH^br^ cells, of which 36.8% were ALDH^very-br^ (pooled sample from 5 hearts). A majority of ALDH^br^ and ALDH^very-br^ cells from young adult hearts stained positive for Sca-1 (*≈*60% and *≈*70%, resp.). Substantial subsets (*≈*25–50%) of ALDH^br^ cells stained positive for CD34, CD106, CD44, CD90, and CD105 (Figure 4). The ALDH^br^ population was significantly enriched for cells expressing Sca-1, CD90, CD34, CD106, and CD44 (fold-changes: 4.2, 4.6, 6.2, 24.1, and 34.3, resp.,) whereas it was significantly depleted for cells expressing CD45 (leukocyte common antigen), CD31 (an endothelial marker), and CD38 (fold-changes: 0.5, 0.36, and 0.22 fold). The immunophenotype of ALDH^br^ cells derived from aging hearts was similar to that of ALDH^br^ cells from young adult hearts.

### 3.4. mRNA Expression of Selected ALDH Isoforms

**Figure 5:**
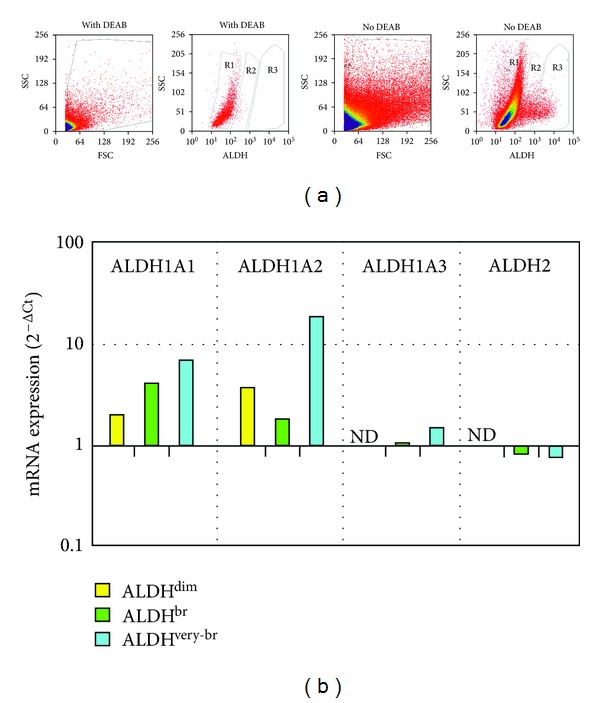
Real time RT-PCR analysis of gene transcripts of selected ALDH isoforms (a). FAC sorting of freshly isolated ALDH^dim⁡^ (R1), ALDH^br^ (R2), and ALDH^very-br^ (R3) atrial cells from 8-week-old mice (b). RT-PCR analysis of ALDH1A1, ALDH1A2, ALDH1A3, and ALDH2 gene transcripts (data are 2^−ΔCt^ values using *GUSB* as a reference gene). ND: not detectable.

mRNA expression of ALDH1A1, ALDH1A2, ALDH1A3, and ALDH2 isoforms was measured by real-time RT-PCR in purified ALDH^dim⁡^, ALDH^br^, and ALDH^very-br^ cells (Figure 5). Gene transcripts of all of the four ALDH isoforms could be detected in ALDH^br^ and ALDH^very-br^ cells, whereas only ALDH1A1 and ALDH1A2 were detectable in ALDH^dim⁡^ cells. In ALDH^br^ and ALDH^very-br^ cells, ALDH1A1 was expressed at higher levels than ALDH1A3 (2^−ΔCt^ = 4.0 and 4.7, resp.,) and ALDH2 (2^−ΔCt^ = 4.8 and 8.8, resp.,) while ALDH1A2 was expressed at higher levels than ALDH1A3 (2^−ΔCt^ = 1.7 and 12.3, resp.,) and ALDH2 (2^−ΔCt^ = 2.1 and 23.2, resp.). In addition, ALDH1A1 was expressed at higher levels in ALDH^br^ and ALDH^very-br^ cells compared to ALDH^dim⁡^ cells (2^−ΔΔCt^ = 2.0 and 3.5, resp.). ALDH1A2 expression was higher in ALDH^very-br^ cells compared to ALDH^dim⁡^ cells (2^−ΔΔCt^ = 4.9) and was lowest in ALDH^br^ cells (2^−ΔΔCt^ = 0.5 versus ALDH^dim⁡^). mRNA expression of three genes implicated in angiogenesis was also measured. 2^−ΔCt^ values for ALDH^dim⁡^, ALDH^br^, and ALDH^very-br^ cells were as follows: endoglin (CD105): 11.70, 10.41, and 14.86; ephrin B4: 2.98, 0.90, and 2.60; angiopoietin 1: undetectable, 0.01, and 0.008, respectively.

### 3.5. *Ex Vivo* Culture-Expansion of ALDH^br^ Cells

**Figure 6:**
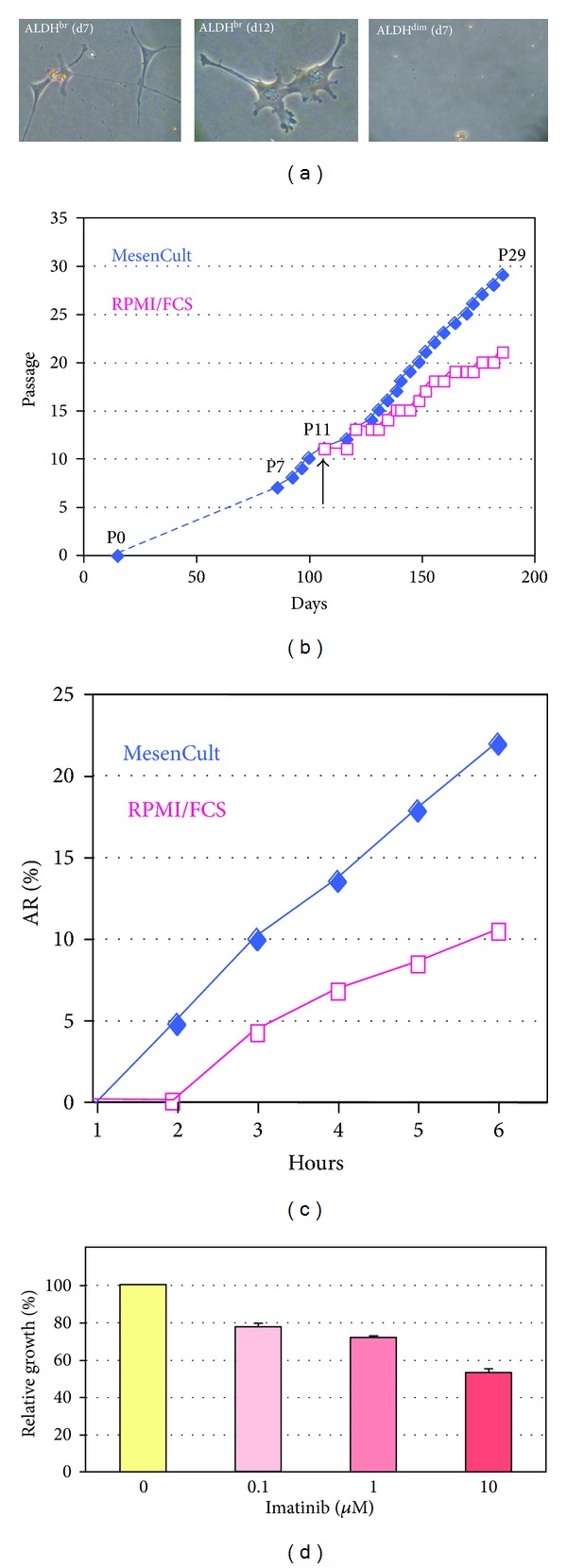
*Ex vivo* culture-expansion of ALDH^br^ atrial cells (a). Photomicrographs of ALDH^br^ cells cultured for 7 and 12 days in MesenCult medium. ALDH^dim⁡^ cells were dead by day 7 (b). Growth curves of cells in different growth media. Cells were cultured in MesenCult medium up to P10 (arrow). From P11 on, MesenCult was either maintained or replaced by RPMI/FCS. Symbols (squares and diamonds) indicate cell passage (no data available from P1 to P7; dotted line) (c). AlamarBlue assay on cells (P40) cultured in either MesenCult or RPMI/FCS (AR%: percent AlamarBlue reduction) (d). Dose-response study of Imatinib with respect to growth inhibition of expanded ALDH^br^ cells (P25).

ALDH^br^ cells were purified from the atrial population, which was enriched for these cells compared to ventricles, in order to abbreviate the FACS procedure, thereby limiting cell damage. Purified atrial ALDH^br^ cells grew in culture, whereas ALDH^dim⁡^ cells did not, even when plated at a 10-fold higher density compared to their ALDH^br^ counterparts (Figure 6(a)). However, >95% ALDH^dim⁡^ cells appeared to be viable, as assessed by DAPI, immediately after the FACS procedure. Atrially derived bulk populations of nonmyocytic cells grew poorly. Purified ALDH^br^ cells gave rise to small numbers of cell colonies, each one apparently originating from a single cell, which formed a monolayer of plastic-adherent cells, which could be expanded for more than 40 passages (later passages were not tested). In one cell culture, the percentage of ALDH^br^ cells at passage 7 was 11%; however, this parameter was not systematically measured at various time points. Growth rates in MesenCult medium were higher than in RPMI/FCS medium (Figure 6(b)). This was confirmed using the AlamarBlue assay (Figure 6(c)). Imatinib inhibited cell growth in a dose-dependent manner (Figure 6(d)). 

### 3.6. Immunophenotype of Culture-Expanded ALDH^br^ Cells

**Figure 7:**
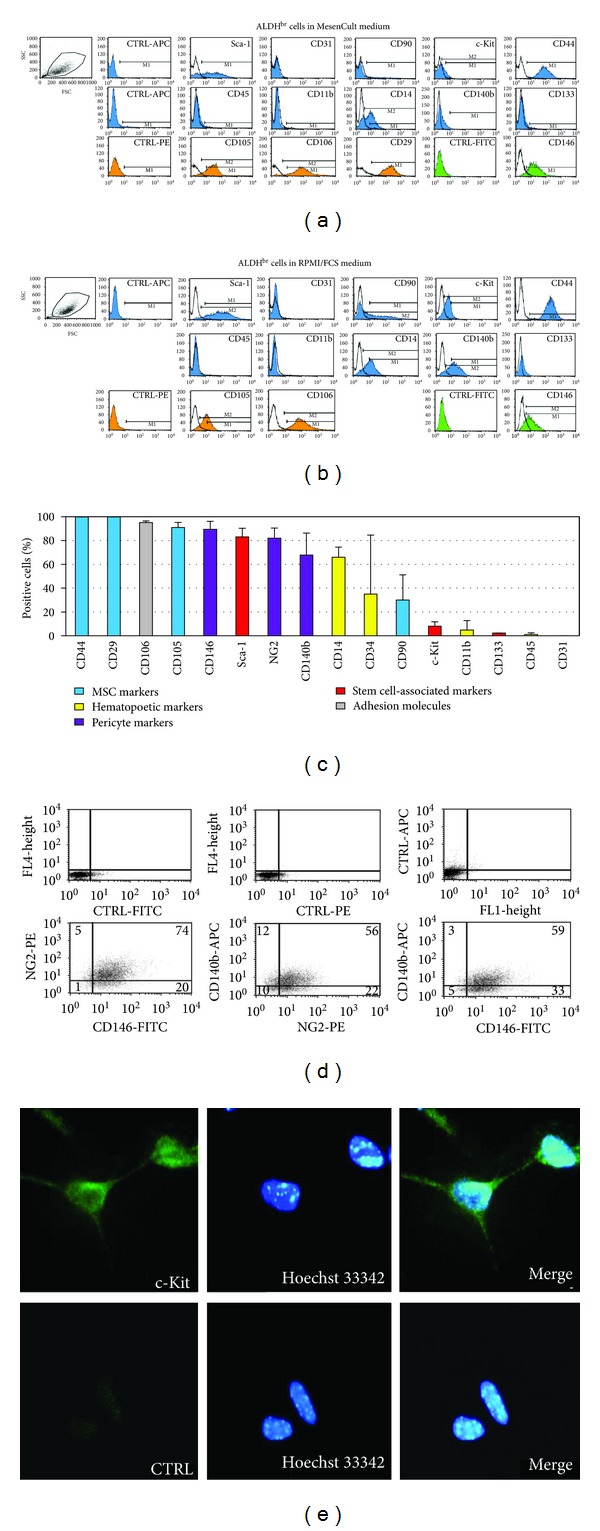
(a)/(b) Flow cytometric analysis of ALDH^br^ atrial cells expanded in MesenCult (P11) or RPMI/FCS medium (P13). (c) Percentages of cells (mean, SD) expressing the indicated markers (data from 3 separate experiments in MesenCult). (d) Two-color analysis of pericyte/perivascular markers (NG2, CD146, and CD140b; numbers are cell percentages). (e) c-kit immunostaining of expanded ALDH^br^ cells. Nuclear staining with Hoechst 33342 (CTRL, secondary Ab only as a control).

The marker profile of expanded ALDH^br^ cells (P11-13) was analyzed by flow cytometry (Figure 7). Cells expanded in MesenCult medium stained positive for Sca-1, CD29, CD44, CD105, CD106, and, in part, CD146 and CD14. They stained negative for CD45, CD11b, CD31, and CD133. To assess whether the culture medium affected marker expression, cells were also grown in RPMI/FCS. The immunophenotype of these cells was similar to those grown in MesenCult, although larger cell subsets stained positive for c-kit, CD140b (PDGFRB) and NG2 chondroitin sulfate proteoglycan (Figures 7(b) and 7(c)). NG2 essentially colocalized with CD140b and CD146 (Figure 7(d)). c-kit expression was demonstrated by immunocytochemistry (Figure 7(e)).

### 3.7. Expanded Cells Express Sarcomeric *α*-Actinin and Differentiate along Mesenchymal Lineages

**Figure 8:**
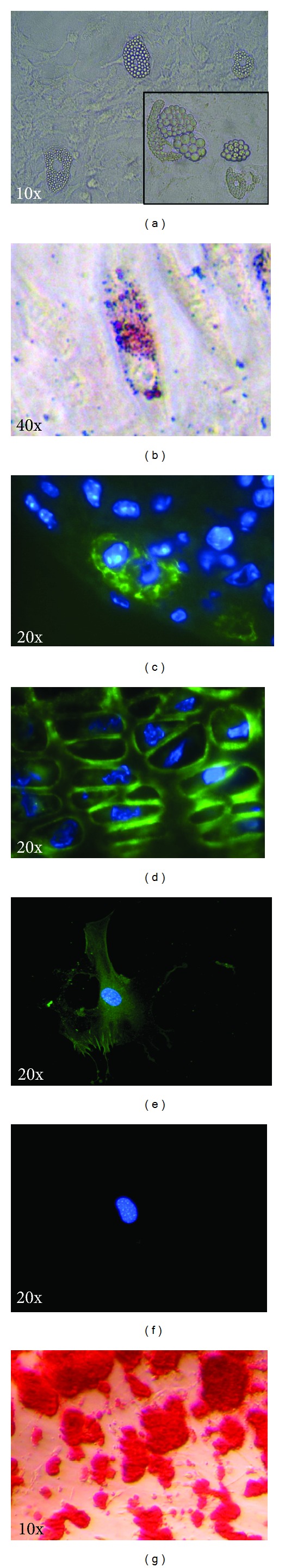
Differentiation potential of expanded ALDH^br^ cells. (a) Photomicrograph showing intracellular fat droplets in cells cultured in adipogenic medium. (b) Positive Oil Red-O staining of intracellular droplets. (c) Positive type-II collagen immunostaining (green) in cells cultured in chondrogenic medium. (d) Type-II collagen immunostaining of leg cartilage, as a positive control. (e) Positive sarcomeric *α*-actinin immunostaining (green) of an ALDH^br^ cell (the protein does not show clear sarcomeric distribution). (f) Corresponding negative control (secondary Ab only). (g) Positive Alizarin Red staining of expanded ALDH^br^ cells cultured in osteogenic medium.

Culture-expanded ALDH^br^ cells could be induced to differentiate along adipogenic, osteogenic, and chondrogenic lineages in appropriate culture media, as evidence by staining with Oil red-O, Alizarin red, Alcian blue (not shown), and type II collagen immunostaining, respectively (Figures 8(a), 8(c) and 8(g)). Cells cultured in MesenCult stained positive for sarcomeric *α*-actinin (Figure 8(e)) but negative for *α*-SMA and von Willebrand factor (not shown).

## 4. Discussion 

Growing evidence suggests high ALDH activity may be a common feature shared by stem and progenitor cells across normal tissues, as well as in cancer. Human UCB and BM cells possessing high ALDH activity have shown angiogenic activity in preclinical studies [30–32] and have been used safely in phase I clinical trials in patients with ischemic cardiovascular disease [33–35].

We characterized, for the first time, ALDH^br^ cells isolated from the heart. Approximately one percent of all nonmyocytic cells present in the young adult mouse heart were ALDH^br^, as measured by Aldefluor stain (Figure 1). The neonatal heart contained a similar number of ALDH^br^ cells, although with a higher proportion of cells exhibiting very high ALDH activity (ALDH^very-br^) compared to the young adult heart. The frequency of ALDH^br^ cells in the population isolated from the atria was approximately 10-fold higher than in cells derived from ventricles (Figure 2). This observation has analogies with previous reports on increased numbers of putative stem/progenitor cells, such as DNA-label retaining cells in rodents [36, 37] and c-kit^+^ cells in human [38], in atria relative to ventricles. In the present study, ALDH^br^ cells isolated from atria and ventricles exhibited slightly different side scatter (SSC) properties. Most atrial ALDH^br^ cells showed SSC^lo^ properties but a small subset possessed intermediate SSC properties. In contrast, ventricular ALDH^br^ cells uniformly exhibited SSC^lo^ properties. The original characterizations of ALDH^br^ cells from human UCB revealed that the ALDH^br^SSC^lo^ population was enriched 50- to 100-fold for primitive hematopoietic progenitors [11, 15]. In several studies, SSC^lo^ properties therefore were associated with the traditional definition of ALDH^br^ hematopoietic cells. However, other studies focused on ALDH^br^Lin^−^ (lineage-depleted) cells [13, 14, 16]. We defined ALDH^br^ cells based solely on ALDH activity, regardless of SSC and lineage properties. This methodological approach affected ALDH^br^ cell counts only marginally, given the predominant ALDH^br^SSC^lo^ phenotype. Nevertheless, the slightly different SSC properties of atrial and ventricular cells may suggest differences in the cellular composition of the ALDH^br^ population depending on the cardiac chamber of origin. This issue remains to be addressed in future studies.

Freshly isolated ALDH^br^ cells exhibited a heterogeneous phenotype, reflecting a mixture of different cell types (Figures 3 and 4). This population was significantly enriched for cells expressing Sca-1, the early hematopoietic and EPC marker CD34, the MSC marker CD90, the hyaluronic acid receptor CD44, and vascular cell adhesion molecule-1 (CD106). CD44 and CD106 were the most highly enriched markers in the ALDH^br^ versus ALDH^dim⁡^ population (34.3 and 24.1 fold-increases, resp.). Previous studies associated CD44 and CD106 expression with MSCs [39]. However, a recent study showed that freshly isolated murine and human BM stromal cells did not express CD44 and CD106, but they could acquire expression of these antigens *in vitro *[40]. CD44^+^CD24^−^ cells have been proposed to represent cancer stem cells in breast cancer [41], as well as in head and neck squamous cell carcinoma (the latter includes an ALDH1^+^CD44^+^CD24^−^ stem cell subset) [42]. In the present study, ALDH^very-br^ cells were highly enriched for Sca-1 and CD90, but only moderately so for CD34, possibly reflecting a predominant MSC-like subpopulation with fewer hematopoietic/vascular progenitors compared to ALDH^br^ cells with lower levels of ALDH activity. The ALDH^br^ population was significantly depleted for common leukocyte antigen (CD45), the endothelial marker CD31, and CD38 (an antigen expressed by the differentiating progeny of CD34^+^CD38^−^ hematopoietic/vascular progenitors). ALDH^very-br^ cells were further depleted for lineage differentiation markers compared to ALDH^br^ cells. Thus, the ALDH^br^ population was enriched for MSC-like progenitors and depleted for differentiating hematopoietic and endothelial cells.

Purified ALDH^br^ atrial cells could be expanded *ex vivo*, whereas their ALDH^dim⁡^ counterparts could not. Because the atrial population contained more ALDH^br^ cells than the ventricular population, atrial cells were used for the purification of ALDH^br^ cells in order to shorten the sorting procedure, and therefore to attenuate cell damage. Purified ALDH^br^ cells formed a few plastic-adherent colonies in each culture well. Each colony apparently originated from a single cell, providing observational evidence of clonal expansion, even though this was not firmly established using a clonogenic assay. The mechanism responsible for the selective growth of ALDH^br^ cells in culture remains unclear. To initially address this question, we determined the respective contributions of selected ALDH isozymes to ALDH activity, measured by Aldefluor stain. Fifteen ALDH isozymes have been described in mice [43] and 19 in human [9, 44]. They play different biological roles that vary among cell types and species. ALDH1A1, the ALDH isozyme most highly overexpressed in human BM ALDH^br^ versus ALDH^dim⁡^ cells [44], was believed to be responsible for ALDH activity, measured by Aldefluor stain, in BM and other tissues. However, recent findings in genetically modified mice demonstrated that ALDH1A1 was dispensable for stem cell function in the mouse hematopoietic and nervous systems [45]. ALDH1 was identified as a marker of normal and malignant human mammary stem cells and a predictor of poor clinical outcome in breast cancer [28]. In a distinct study, shRNA knock-down data indicated that ALDH1A3, not ALDH1A1, correlated best with ALDH activity in breast cancer stem cells [46]. ALDH1A1 and ALDH3A1 were highly expressed in nonsmall cell lung cancer, and knock-down of these ALDH isoforms were associated with *in vitro* functional changes in the proliferation and motility of these cells [47]. Human ALDH1A1, ALDH1A2, ALDH1A3, and ALDH8A1 function in retinoic acid cell signaling via retinoic acid production by oxidation of *all-trans*-retinal and 9*-cis*-retinal [6, 46]. ALDH2, a mitochondrial isozyme that mediates both the detoxification of reactive aldehydes and the bioactivation of nitroglycerin to nitric oxide, mediates cytoprotection in the heart [48]. Recent data indicated that the aldehyde-oxidizing activity of mouse HSCs, measured by Aldefluor stain, was due to the ALDH2 isozyme and correlated with protection against acetaldehyde toxicity [49]. Mice deficient in both Fanconi anemia pathway-mediated DNA repair and acetaldehyde detoxification showed more than a 600-fold reduction in the HSC pool.

Based on these reports, we measured the expression of ALDH1A1, ALDH1A2, ALDH1A3, and ALDH2 gene transcripts in purified ALDH^dim⁡^, ALDH^br^, and ALDH^very-br^ cardiac-derived cells (Figure 5). Expression of ALDH1A1 was lowest in ALDH^dim⁡^ cells, intermediate in ALDH^br^ cells, and highest in ALDH^very-br^ cells (2^−ΔΔCt^ = 3.5 using ALDH^dim⁡^ as a reference population). By contrast, expression of ALDH1A2 was lowest in ALDH^br^ cells, intermediate in ALDH^dim⁡^ cells, and highest in ALDH^very-br^ cells (2^−ΔΔCt^ = 4.9 versus ALDH^dim⁡^). Expression of ALDH1A3 and ALDH2 was detectable in ALDH^br^ and ALDH^very-br^ cells, albeit at lower levels compared to ALDH1A1 and ALDH1A2, but not in ALDH^dim⁡^ cells. Thus, ALDH1A1 correlated best with ALDH activity; however, contributory roles of ALDH1A2, ALDH1A3, and ALDH2 to ALDH activity are possible based on our data. While the present analysis included the four ALDH isoforms most often associated with ALDH activity in previous reports [44–49], it should be completed to include the remaining ALDH isoforms. In addition, shRNA knock-down experiments are needed for identifying the ALDH isozyme(s) responsible for ALDH activity in these cells. 

Recent data suggested BM and UCB ALDH^br^ cells might repair ischemic tissues *in vivo* by releasing angiogenic factors [30–32]. A comparison of gene expression profiles by ALDH^br^ and ALDH^dim⁡^ human BM cells identified three angiogenic factors (endoglin/CD105, ephrin B4, and angiopoietin-1) as the most highly overexpressed factors in ALDH^br^ versus ALDH^dim⁡^ cells (fold-changes: 66.9, 64.6, and 7.0, resp.) [44]. We therefore measured mRNA expression of these angiogenic genes in cardiac-derived cells but found no correlation with ALDH activity (besides the preliminary observation that angiopoietin-1 transcripts were detectable in ALDH^br^ and ALDH^very-br^ cells but not in ALDH^dim⁡^ cells). The angiogenic potential of cardiac-derived ALDH^br^ cells remains to be addressed in future studies. Previous reports showed that ALDH^br^ MSCs from human UCB were more responsive to hypoxia than their ALDH^dim⁡^ counterparts, with upregulation of Flt-1, CXCR4, and angiopoietin-2 [50]. The same group reported that ALDH^dim⁡^ EPCs, but not ALDH^br^ EPCs, from human UCB upregulated hypoxia-inducible factor proteins as well as VEGF, CXCR4, and GLUT-1 mRNAs under hypoxic conditions [22]. The introduction of ALDH^dim⁡^ EPCs significantly reduced ischemic tissue damage in a mouse flap model, whereas ALDH^br^ EPCs were ineffective. These findings suggested varying angiogenic activities of ALDH^br^ cells depending on the cell type studied.

In the present study, plastic-adherent cell clusters grew rapidly and could be passaged more than 40 times (later passages were not tested). Growth rates in complete MesenCult medium were higher than in RPMI/FCS (Figure 6). Imatinib, which selectively inhibits PDGFRB (CD140b) and c-kit tyrosine kinases with a similar potency [51], reduced cell proliferation in a dose-dependent manner. Expression of PDGFRB and c-kit was detected in subsets of expanded cells. The effect of Imatinib might reflect a role for PDGFRB and/or c-kit in the proliferation of these cells. Previous studies showed that ALDH^br^ MSCs from UCB proliferated more than their ALDH^dim⁡^ counterparts [50], whereas ALDH^dim⁡^ EPCs proliferated more than ALDH^br^ EPCs [22]. Proliferative properties correlated with angiogenic activities in these populations. Again, the association of ALDH and proliferation may vary among cell types. The immunophenotype of expanded ALDH^br^ cardiac cells resembled that of freshly isolated ALDH^br^ cells (Figure 7). Positive stains for pericytic markers (NG2, CD146, and CD140b) were consistent with a MSC-like phenotype. It has been shown that perivascular cells of arterioles and capillaries from many tissues express pericytic markers and show MSC-like and angiogenic features [52–54]. In the present study, expanded cells stained positive for sarcomeric *α*-actinin, a cardiac marker, and exhibited differentiation potential along multiple mesenchymal lineages. Previous data showed that aldehyde dehydrogenase activity did not increase the chondrogenic potential of human adipose-derived adult stem cells [55]. Recent reports showed that human BM contained ALDH^br^ multipotential mesenchymal progenitor cells (MPCs), besides ALDH^br^ HSCs and EPCs [12, 56]. These MPCs strongly expressed ALDH, expressed embryonic markers not present in mesenchymal cells, and could be differentiated to microvascular endothelial cells and mesenchymal cells. The latter lacked strong ALDH expression and could not be induced to back differentiate into MPCs.

We observed a loss of ALDH^br^ cells with cell passage (e.g., only 11% of cells at passage 7 were still ALDH^br^). Whether this loss of ALDH^br^ cells with time reflected cellular adaptation to *ex vivo* conditions or the replacement of early progenitors by a progeny of late progenitor and precursor cells remains unclear. The observation that growth rates and the immunophenotype remained stable for more than 30 and 10 passages, respectively, seemed not to support cell differentiation. ALDH as a stem/progenitor cell marker has chiefly been used in freshly isolated cells and tissues, as opposed to expanded cell populations. The significance of ALDH in culture-expanded cells is unclear, as is its contribution to stem cell fate. The aforementioned observation that the mesenchymal cell progeny of ALDH^br^ human BM cells lacked strong ALDH expression and could not be induced to back differentiate into MPCs suggested a parallel loss of ALDH expression and cell potency [56].

In conclusion, cardiac-derived ALDH^br^ cells are progenitor cells with MSC-like phenotype and function and with superior *ex vivo* growth characteristics compared to their ALDH^dim⁡^ counterparts. Further studies are needed for assessing the regenerative potential of these cells in animal models of heart disease.
